# 

**Published:** 1899-09-30

**Authors:** 


### NOTICE TO CORRESPONDENTS.

All MS., Letters, Books for Review, and other matters intended for the Editor should be addressed The Editor, "The Hospital," The Hospital Building, 28 & 29, Southampton Street, Strand, W.C.

The Editor cannot undertake to return rejected MS., even when accompanied by stamped directed envelope.

**Notice to Advertisers and Subscribers.**

All Advertisements, Orders for Copies of The Hospital, and Business Communications should be addressed to **THE MANAGER (Not to the Editor),** The Hospital, The Hospital Building, 28 & 29, Southampton Street, Strand, London, W.C.

The Cost of Subscription, post free, is as follows:—Per week, 2½d.; per quarter, 2s. 8d.; per half-year, 5s. 3d.; per annum, 10s. 6d. Foreign:—Per annum, 15s. 2d., payable, in all cases, in advance.

**NOTICE.—Vol. XXV. of "The Hospital" (October, 1898, to March, 1899), in handsome cloth binding, is now on sale at the Office of this Journal, price 7s. 6d. Cases for binding the Numbers contained in this Volume 1s. 6d. Index to Vol. XXV., 2½d., post free.**

**The Monthly Part for October will be ready on October 2nd. Price 6d., by post 8d.**

### BOOKS RECEIVED.

The Scientific Press.

"Burdett's Hospitals and Charities," 1899. By Sir Henry Burdett, K.C.B.

"A Handbook for Nurses." By J. K. Watson, M.D.

Baillière, Tindall, and Cox.

"Urinary Analysis and Diagnosis." By Louis Hertzmann, M.D.

Whittaker and Co.

"A Dictionary of Terms used in Medicine." By the late Richard D. Hoblyn, M.A. Thirteenth edition. Revised, with numerous additions, by John A. P. Price, B.A., M.D.

Gale and Polden.

"Strength, and How to Obtain It." By Eugen Sandow.

**Periodicals and Pamphlets.**—Sanitary Record, Municipal Journal, and London Medical Record, Medical Press, Sunday at Home.

### The Student of Medicine.

### APPOINTMENTS.

**Louisa Garrett Anderson, M.B., B.S.Lond., appointed Junior Assistant Medical Officer at the Camberwell Infirmary. G. Butter, M.R.C.S.Eng., appointed House Surgeon to the Zeehan Hospital, Tasmania. W. T. Davies, M.R.C.S., L.R.C.P., appointed Medical Officer for the Llanbister District of the Knighton Union. C. E. Forsyth, M.B., Ch.M.Aberd., appointed Assistant Medical Officer, County Asylum, Dorchester. H. Horrocks, M.D., B.Sc.Lond., M.R.C.S., L.R.C.P., D.P.H.Lond., appointed a member of the Dental Board of Western Australia. G. Hungerford, L.R.C.P.&L.R.C.S.I., appointed Assistant Medical Officer to the Wonford Hospital for the Insane, Exeter. Madge S. Maclean, M.B., C.M.Glasg., appointed House Surgeon to the Victoria Hospital for Sick Children, Hull. J. P. Milton, M.R.C S., L R.C.P.Lond., appointed Medical Officer for the No. 5 District of the Penzance Union. E. W. Ormerod, M.R.C.S., L.R.C.P.Lond., appointed Medical Officer to the Southam Union Workhouse. J. S. Park, L.R.C.P., appointed Health Officer for Cranbourne Shire, Victoria. S. H. Rentzsch, M.R C.S.Eng., L.R.C.P.Lond., appointed Medical Officer for the South District of the Stratton Union. E. Rommeis, M.D., appointed Health Officer for North Freemantle, Western Australia. C. P Stewart, M.B., M.S, B.Sc.Edin., appointed Medical Officer of Health and Police Surgeon for Perth. T. Wilson, M.B., appointed Resident Medical Officer for the Katanning District and Public Vaccinator for Katanning, Western Australia.**

**BURDETT'S HOSPITALS AND CHARITIES, 1899.**

